# GamReg Sweden—Protocol for a systematic cohort data collection for improved clinical knowledge in specialized gambling disorder treatment

**DOI:** 10.3389/fpsyt.2022.894532

**Published:** 2022-09-12

**Authors:** Anders Håkansson, Gunny Åkesson

**Affiliations:** ^1^Gambling Disorder Unit, Region Skåne, Malmö Addiction Center, Malmö, Sweden; ^2^Department of Clinical Sciences Lund, Psychiatry, Faculty of Medicine, Lund University, Lund, Sweden

**Keywords:** gambling disorder, quality register, behavioral addiction, problem gambling, gaming disorder

## Abstract

**Background:**

Gambling disorder is the first non-substance-related addiction which is recognized as a diagnostic entity and assessed in treatment settings. However, in many clinical settings, assessment, and structured treatment for this condition is severely under-developed, and treatment seeking in many settings is low. This is a protocol paper describing the rationale and structure of a recently established quality register, allowing for structured monitoring of treatment seeking, treatment needs and treatment provision in Swedish health care settings, for gambling disorder and associated conditions.

**Methods:**

Since 2019, a Swedish quality register is in use for the systematic data collection from patients receiving treatment in specialized health care. The register is held by Region Skåne, and approved for national use. Swedish quality registers allow for the clinical monitoring of treatment uptake and needs, for quality improvement purposes, and collect systematic cohort data for these purposes. In addition, these quality registers potentially allow for future research projects, after separate ethics applications, allowing for clinical follow-up studies based on non-identified quality register data. Clinical challenges and research knowledge gaps are addressed in the present register, including mental health comorbidity, history of suicidal behavior, comorbid alcohol, drugs and gaming behaviors, and fundamental psycho-social variables such as violence victimization, concerned significant others including children's situation in families of problem gamblers, and main income and involvements with social services and enforcement agency. In addition, patient flows, including rates of referral from primary care and other treatment settings, can be followed. The overall quality register project is registered at clinicaltrials.gov (NCT05276193).

**Discussion:**

The present protocol paper will allow for systematic reporting and future projects addressing knowledge gaps in clinical treatment for gambling disorder, and highlight the importance for evidence-based treatment in a behavioral addiction. Importantly, the current data will contribute to a better understanding of which patient groups may be less likely to seek or to be referred to treatment, and thereby may shape future initiatives to increase screening and referral in targeted, vulnerable groups.

## Introduction

Gambling disorder is a condition recognized by diagnostic manuals, and representing the first addictive disorder not involving a substance (1), and which causes a range of severe public health consequences (2). Although treatment recommendations for this condition are relatively clear, primarily involving cognitive-behavioral therapy and/or motivational interviewing (3), treatment seeking tends to be low (4), due to limited treatment provision or other barriers perceived by individuals affected by this condition.

Unlike substance-related addictive disorders, diagnostic and therapeutic interventions for gambling disorder are therefore often not fully developed or integrated in health care services where addictive disorders and mental illness are assessed and treated. In Sweden, a setting where regional health care providers and local municipality social services share the responsibility for addiction treatment, gambling disorder was for many years not included in routine assessment and treatment for addictive disorders. While gambling was formally not included in the legislation regulating social services until 2018, treatment uptake for gambling disorder was very limited in the Swedish health care system, with a very low number of patients seen annually (5). Apart from a limited number of local treatment initiatives, self-help groups instead played an important role in providing informal support for patients with gambling problems and for their concerned significant others (6).

Thus, clinical uptake of gambling disorder in the present setting is limited; the number of patients treated in out-patient specialized settings for alcohol use disorders was around 40 times higher than for gambling (7). Therefore, clinical experience is likely to be markedly more limited for gambling than for substance use disorders. In addition, there is reason to believe that many people affected by gambling problems do not seek treatment, or do not perceive public treatment institutions to be able to address gambling problems as well as they would for other addictive or mental health disorders. For example, a web survey in the present setting demonstrated that only 50 percent of respondents from the general population primarily thought of a public institution as a natural way to seek treatment for this condition, while 50 percent primarily thought of a self-help group outside of the official treatment system (8). Likewise, there is need to monitor how treatment seeking, and characteristics of attending patients, may change over time. For this reason, the present protocol paper describes the development and implementation of an official quality register for developmental purposes related to treatment of gambling disorder in the Swedish health care system, and which will allow for longitudinal descriptive and follow-up projects.

## Methods

### Setting

The present register is held by the first author and administered by the public institution Register center South (Registercentrum Syd (9)), associated with the health care services in southern Sweden. The application (to the administrative board of Region Skåne) to establish the register was formally approved in February, 2018 (Region Skåne, file number 221-17). An amendment, adding the possible purpose also to register treatment contacts related primarily to problem gaming instead of problem gambling, was approved in December, 2018. The protocol of the register project has been registered at clinicaltrials.gov (NCT05276193). The establishment of Swedish health care quality registers does not require ethical permission, whereas each separate future research project conducted on the material requires such permission (7, 9–11). The establishment of a quality register in Swedish health care is regulated by a national legislation and applications and local administration are monitored by regional health care organizations (11).

### Procedure

Currently, one region (Region Skåne) systematically registers in the present quality register. This registration is carried out by the regional gambling disorder treatment facility of Region Skåne (one of the major regions, including one of the three main urban areas of Sweden), under the governance of the clinical facility of Malmö Addiction Center (Competence Center Addiction, Region Skåne, Malmö, Sweden). Any health care region in Sweden will have the opportunity to adhere to the quality register, and to register treatment contacts involving problem gambling or problem gaming. At the facility owning the register and where registration has started, ~10 patients seek treatment per month, with typically around 25–30 patients in active contact per month. Thus, the number of patients to be registered can expand substantially with the number of health care regions who adhere to the register.

Swedish quality registers are based on an opt-out consent procedure, where individuals in contact with the relevant health care facilities are informed about the register procedures in a standardized way, and with the possibility to opt out from any registration (through a telephone or written contact, or through a personal message to staff of the facility). After a trial period in which applying patients were informed verbally within the treatment contact, patients are now systematically informed (since August 27, 2020) by information letter when they apply to or are referred to the facility.

After the termination of a treatment contact, the variables of the register are completed in a confidential (two-factor coded access) electronic database. A CBT-trained therapist and research assistant (such as the second author of the present paper), carries out the registration, based on available health care documentation from the treatment episode. Data to be registered rely on clinical information originating from the treatment episode carried out. The data registered are based on the content of the patient's health care documentation at the unit (typically from structured assessment at the start of the treatment episode, documented by a physician and by the therapist, along with additional information from subsequent therapy visits). Thus, no self-report procedure is carried out for the registration. Patients are registered with their personal and unique social security number (“person number”), and patients with more than one treatment episode can be registered more than once, and their separate treatment occasions can be identified separately, including for follow-up purposes or in order to exclude duplicates.

### Register variables

Several key components of clinical assessment in gambling disorder are systematically included in the register. The choice of variables to include in the register, and the wording of these variables on the data sheet used for data collection and registration, were discussed and reviewed within the steering group of the register, which consists of individuals with long-standing experience of health care work with gambling and other addictive disorders, social work in the municipal setting, issues related to children and concerned significant others of patients with addictive disorders, and representatives with a personal history of gambling problems. The members of this group were affiliated with the health care services in Skåne Region and the Gothenburg Region, the city of Malmö social services, and a major patient's and peer support organization for patients with gambling problems and their families (6).

These variables include (1) date of the initiation of the treatment contact, the patient's gender, age, and occupation, (2) age of onset of problem gambling, (3) types of gambling associated with the present treatment contact, (4) way of referral to the present treatment contact, (5) contact with mental health care, social services, and enforcement agency, (6) history of suicidal acts, (7) alcohol and drugs problems requiring assessment or treatment, (8) central stimulant medications, (9) violence victimization, (10) living conditions including the responsibility for underage children, and the facility's offered interventions for the patient's children, (11) treatment methods chosen for the present treatment contact, and (12) potential addictive pattern related to video gaming, including a treatment need for gaming disorder.

Specifically, each of the main domains assessed in the register is explained by a clinical research and developmental knowledge gap identified, or by a clinical need:

#### Age and gender

Extensive gender differences have been seen between female and male problem gamblers, both with respect to the types of gambling associated with problems, and with a different time course which is closely associated with a marked age difference between male and female patients. In addition, gambling problems appear to commence at different ages in life in women and men (12). Also, it has been argued that women with gambling problems may face barriers against seeking and receiving treatment, which underlines the need to monitor gender and age of patients seen in treatment. In the present setting, as in other countries, a majority of treatment-seeking patients are men, although there are signs indicating that the proportion of women among problem gamblers is increasing (13).

#### Type of gambling

While a broad spectrum of gambling types appear on the gambling market, some gambling types may be considerably more common than others in patients with disordered gambling (14), and the distribution of such gambling types may differ over time and across different geographical settings. When relevant, both online and land-based gambling types are included in the data sheet underlying the reporting to the register. The gambling types included in the report sheet include sports betting (live and non-live), casino, electronic gambling machines, bingo, horse race betting, lotteries, number games, card games including poker, and gambling for money within videogames. Importantly, the gambling types registered may not only include traditional land-based and online-based gambling types, but also stock market trading (15).

#### Other treatment and institutional contacts

Mental health comorbidity, including comorbid alcohol and drug use disorders, is common in gambling disorder (16), as well as suicidal behavior, both expressed as suicide attempts (17) and suicidal ideation (18). In addition, over-indebtedness is common in gamblers (19), and being indebted is known to be associated with suicidal behavior (20). Likewise, one of the most common conditions assessed and treated in Swedish specialized psychiatry is attention-deficit/hyperactivity disorder (ADHD), for which central stimulant medications are typically prescribed. Importantly, a markedly increased risk for problem gambling has been documented in individuals with attention-deficit/hyperactivity disorder (ADHD (21)), highlighting the need to assess this comorbidity among treatment-seeking patients with problem gambling.

#### Violence victimization

Any history of psychological, physical or sexual violence victimization will be reported. A link between gambling problems and with a history of this type of traumatic events previously has been suggested, although insufficiently outlined and followed in clinical settings (22).

#### Gaming behavior

In parallel with the establishment of this systematic quality register for gambling disorder, treatment occasions which partly or primarily address problem gaming will also be possible to register. While such clinical interventions are even less developed than those related to gambling, the gaming disorder diagnosis receives increasing scientific and clinical attention, as it has formally been established as a behavioral addiction by the World Health Organization in 2018 (23). Prevalence of gaming disorder is hitherto insufficiently described, with wide variability across studies and countries (24), but a likely association between problem gaming and problem gambling has been suggested to occur in some patients, although it is also clear that the conditions represent conceptually different entities (25). In an additional item of the present register, it is noted if the treatment contact involved a problematic gaming behavior, and whether the type of gaming behavior assessed referred to a single-player, multiplayer, or massively multiplayer online game, or other.

#### Referral to the present treatment contact

The way of referral to the facility, or the independent treatment-seeking behavior of an individual making a self-referral, is of importance to monitor. This may reflect both the actual treatment seeking of the population (8), and the treatment uptake in other parts of the health care service, such as in primary care, which has been pointed out as an important arena for the screening and referral for problem gambling (26). Other points of referral include, among others self-help groups, social services, and the criminal justice system.

#### Living conditions including the responsibility for underage children

The problematic situation of children of problem gamblers has been increasingly highlighted (27). In the present register, it is indicated whether the patient lives with a partner, whether the patient lives with any children under the age of 18 years, and whether the child/children have received any type of counseling or support during the treatment contact.

#### Treatment methods

The treatment method, or methods, used during the treatment contact is registered. This may involve the more typically recommended treatments, such as cognitive behavioral therapy (delivered individually, in group, or online) or motivational interviewing, or pharmacological treatment such as naltrexone or nalmefene (3). Also, other possible options include 12-step-oriented treatment, psychodynamic treatment, or residential treatment.

## Discussion

This is a protocol paper describing the rationale and structure of a quality register aimed to improve clinical understanding and structured follow-up and outcome in gambling disorder. Being a novel condition in many clinical settings, despite its long-standing history as an addiction recognized in the community, the treatment of gambling disorder in many settings might require the development of clinical knowledge and systematic cohort data, allowing for longitudinal follow-up of which patient groups are seen, and their comorbidities. Likewise, unlike typical health care registers, the present type of data collection also can give rise to the study of children and concerned significant others in relation to gambling problems. In the present setting, few health care institutions have a long history of treating gambling disorder (14), and in this and other settings, voluntary treatment seeking in patients with gambling problems is known to be a challenge (4, 8).

The understanding of treatment seeking and treatment provision for this disorder will require systematic data collection, which is rarely carried out for behavioral addictions. Building major register databases, for continuous data collection, can provide large-scale data of value for a number of issues difficult to study in smaller, study-specific cohorts. One example is the US veteran databases which have been able to provide key data in problem gambling and other addictive disorders over many years (28). The establishment of the present official quality register will allow for the systematic reporting of treatment seeking, treatment content and key feature of patients seen in treatment. The present type of data will contribute to a better understanding of which patient groups may be less likely to seek or to be referred to treatment; thereby, data originating from this type of dataset may shape future initiatives to optimize treatment content and target screening in vulnerable groups. For example, the extent of online or land-based gambling types in treatment-seeking patients can be monitored and followed over time. Future development and research projects can address knowledge gaps in clinical treatment for gambling disorder, using the present register for non-identified baseline cohort data, and for similar purposes where registers are run against each other in order to identify key variables in gambling disorder treatment and follow-up. While the duration or frequency of treatment, and the details and diagnoses from parallel or subsequent treatments of comorbidity are not included in the register, the combination of the present register (after relevant permission procedures and ethics approval) can be carried out and may contribute to a further understanding of clinical aspects outside of the present facility's key data. Both psychiatric and physical disorders are thereby available for analysis from national patient registers, as described in previous register-based work form the present setting (5). Such combined use of registers, in specific research projects, may also involve registers of prescription drugs available in the present setting, allowing for in-depth knowledge on comorbidities. This may, for example, include the type of dopaminergic compounds (typically dopamine agonists in Parkinson's disease or restless legs syndrome) suspected to cause or increase problem gambling in a sub-group of patients (29).

## Limitations

As the present register aims to include nearly a totality of patients receiving treatment at the units included, for the sake of brevity and efficacy in the everyday working situation, only data reported by staff from the treatment episodes are included, rather than self-report questionnaires of similar. For this reason, for example, a quantitative measure of gambling disorder severity cannot be included in the quality register data included here. Other such examples include items about other types of problem behaviors, such as other addictive online behaviors or other behavioral addictive behaviors, which cannot be covered by the present type of structured and intentionally brief documentation-based data collection. Thus, in light of the need for implementation of a systematic data collection and a framework for quality and research projects, the reduced detail level in the data is an inherent limitation of this type of register. This may, for example, include the lack of a severity assessment. Also, as a necessary consequence of the present establishment of a structured clinical data source, the present type of health care quality register cannot provide data on non-treatment-seeking patients with gambling disorder. For this type of analyses, we rely on general population surveys (8, 13, 19).

## Ethics and dissemination

For the registration and initiation of a Swedish quality register (7), no ethical permission from a national ethics authority is required. However, the establishment of the register was approved by the relevant authority (the health care region of Skåne, Sweden). Also, the establishment and aim of the register are made public through an online website in original language (Swedish, Registercentrum Syd (9)) and registered internationally and openly on clinicaltrials.gov (NCT05276193). Patients can opt out from registration in health care quality register of the present type in Sweden. No data which can be linked to an identified individual is published from the register. However, any future scientific evaluation of the data in the register requires such permissions, specifically for each research project. The format of Swedish quality registries, their role as a potential basis for future research projects, and the need for ethical permissions upon such future projects, have been described in the literature (10).

The steering group of the register contains representatives from a national peer support organization for patients with problem gambling, and for their relatives. Data from the quality assessment of the register, including annual changes in the distribution of treatment-seeking patients, will be disseminated in public and to policy makers and peer support organizations regularly.

## Ethics statement

For the registration and initiation of a Swedish quality register, no ethical permission from a national ethics authority is required. However, the establishment of the register was approved by the relevant authority (the health care region of Skåne, Sweden). Also, the establishment and aim of the register are made public through an online website in original language (Swedish, Registercentrum Syd, 2020) and registered internationally and openly on clinicaltrials.gov (NCT05276193). Patients can opt out from registration in health care quality register of the present type in Sweden.

## Author contributions

AH and GA are responsible of the overall idea and design of the paper and the project. GÅ is responsible of data registration, and AH is the main responsible of the overall project. AH wrote the first draft of the paper, and GÅ reviewed and edited it. Both authors approved and are responsible of the final version of the paper.

## Funding

The research group has overall funding from the state-owned gambling operator of Sweden (AB Svenska Spel), from the research councils of Svenska Spel and the alcohol monopoly Systembolaget AB, from the Region Skåne hospital organization, and from the office of the southern Swedish health care regions. None of these organizations were directly involved in the present paper.

## Conflict of interest

The research group has overall funding from the state-owned gambling operator of Sweden, AB Svenska Spel, and from the research council of AB Svenska Spel, the research council of the alcohol monopoly Systembolaget AB, and the hospital regions of southern Sweden including Region Skïne. None of these organizations had any influence on the present paper.

## Publisher's note

All claims expressed in this article are solely those of the authors and do not necessarily represent those of their affiliated organizations, or those of the publisher, the editors and the reviewers. Any product that may be evaluated in this article, or claim that may be made by its manufacturer, is not guaranteed or endorsed by the publisher.

## References

[1] Potenza MN Balodis IM Derevensky J Grant JE Petry NM Verdejo-Garcia A . Gambling disorder. Nat Rev Dis Prim. (2019) 5:51. 10.1038/s41572-019-0099-7 31346179

[2] Reith G Wardle H Gilmore I. Gambling harm: a global problem requiring global solutions. Lancet. (2019) 394:1212–4. 10.1016/S0140-6736(19)31991-9 31443927

[3] Di Nicola M De Creszenzo F D'Alò GL Remondi C Panaccione I Moccia L . Pharmacological and psychosocial treatment of adults with gambling disorder: a meta-review. J Addict Med. (2020) 14:e15–23. 10.1097/ADM.0000000000000574 31651561

[4] Gainsbury S Hing N Suhonen N. Professional help-seeking for gambling problems: awareness, barriers and motivators for treatment. J Gambl Stud. (2014) 30:503–19. 10.1007/s10899-013-9373-x 23494244

[5] Hakansson A Karlsson A Widinghoff C. Primary and secondary diagnoses of gambling disorder and psychiatric comorbidity in the Swedish health care system – a nationwide register study. Front Psychiatry. (2018) 9:426. 10.3389/fpsyt.2018.00426 30258370 PMC6143815

[6] Binde P. A Swedish mutual support society of problem gamblers. Int J Mental Health Addict. (2012) 10:512–23. 10.1007/s11469-011-9335-4

[7] Swedish Board of Health and Welfare. [*Substance Misuse, Substance-Related Diagnoses, and Gambling*]. Missbruk, substansrelaterade diagnoser och spel om pengar. (English summary). Stockholm: Swedish Board of Health and Welfare (2021).

[8] Håkansson A Ford M. The general population's view on where to seek treatment for gambling disorder – a general population survey. Psychol Res Behav Manag. (2019) 12:1137–46. 10.2147/PRBM.S226982 31908551 PMC6927584

[9] Registercentrum Syd. GamReg Sweden. Available online at: https://rcsyd.se/anslutna-register/gamreg-sweden Registercentrum Syd, Lund, Sweden (2022).

[10] Emilsson L Lindahl B Köster M Lambe M Ludvigsson JF. Review of 103 Swedish healthcare quality registries. J Intern Med. (2015) 277:94–136. 10.1111/joim.12303 25174800

[11] Swedish Association of Local Authorities and Regions. Quality registries. Available online at: https://skr.se/en/kvalitetsregister/omnationellakvalitetsregister.52218.html Swedish Association of Local Authorities and Regions (2021).

[12] Zakiniaeiz Y Cosgrove KP Mazure CM Potenza MN. Does telescoping exist in male and female gamblers? Does it matter? Front Psychol. (2017) 8:1510. 10.3389/fpsyg.2017.01510 28928697 PMC5591942

[13] Abbott MW Romild U Volberg RA. The prevalence, incidence, and gender and age-specific incidence of problem gambling: results of the Swedish longitudinal gambling study (Swelogs). Addiction. (2018) 113:699–707. 10.1111/add.14083 29105942

[14] Håkansson A Mårdhed E Zaar M. Who seeks treatment when medicine opens the door to gambling disorder patients – psychiatric co-morbidity and heavy predominance of online gambling. Front Psychiatry. (2017) 8:255. 10.3389/fpsyt.2017.00255 29238309 PMC5712539

[15] Arthur JN Delfabbro P. Day traders in South Australia: similarities and differences with traditional gamblers. J Gambl Stud. (2017) 33::855–66. 10.1007/s10899-016-9659-x 27988861

[16] Dowling NA Cowlishaw S Jackson AC Merkouris SS Francis KL Christensen DR. Prevalence of psychiatric co-morbidity in treatment-seeking problem gamblers: a systematic review and meta-analysis. Austr N Z J Psychiatry. (2015) 49:519–39. 10.1177/0004867415575774 25735959 PMC4438101

[17] Håkansson A Karlsson A. Suicide attempt in patients with gambling disorder – associations with comorbidity including substance use disorders. Front Psychiatry. (2020) 11:593533. 10.3389/fpsyt.2020.593533 33304287 PMC7701043

[18] Ronzitti S Soldini E Smith N Potenza MN Clerici M Bowden-Jones H. Current suicidal ideation in treatment-seeking individuals in the United Kingdom with gambling problems. Addict Behav. (2017) 74:33–40. 10.1016/j.addbeh.2017.05.032 28570912

[19] Håkansson A Widinghoff C. Over-indebtedness and problem gambling in a general population sample of online gamblers. Front Psychiatry. (2020) 11:7. 10.3389/fpsyt.2020.00007 32116832 PMC7016486

[20] Rojas Y. Financial indebtedness and suicide: a 1-year follow-up study of a population registered at the Swedish Enforcement Authority. Int J Soc Psychiatry. (2021) 2:207640211036166. 10.1177/00207640211036166 34340574 PMC9548947

[21] Jacob L Haro JM Koyanagi A. Relationship between attention-deficit hyperactivity disorder symptoms and problem gambling: a mediation analysis of influential factors among 7,403 individuals from the UK. J Behav Addict. (2018) 7:781–91. 10.1556/2006.7.2018.72 30238788 PMC6426384

[22] Dowling N Suomi A Jackson A Lavis T Patford J Cockman S . Problem gambling and intimate partner violence: a systematic review and meta-analysis. Trauma Violence Abuse. (2016) 17:43–61. 10.1177/1524838014561269 25477014

[23] World Health Organization. Gaming disorder. Available online at: https://www.who.int/features/qa/gaming-disorder/en/ World Health Organization. (2018).

[24] Stevens MW Dorstyn D Delfabbro PH King DL. Global prevalence of gaming disorder: a systematic review and meta-analysis. Aust N Z J Psychiatry. (2021) 55:553–68. 10.1177/0004867420962851 33028074

[25] Mallorquí-Bagué N Fernández-Aranda F Lozano-Madrid M Granero R Mestre-Bach G Baño M . Internet gaming disorder and online gambling disorder: clinical and personality correlates. J Behav Addict. (2017) 6:669–77. 10.1556/2006.6.2017.078 29280393 PMC6034948

[26] Achab S Chatton A Khan R Thorens G Penzenstadler L Zullino D . Early detection of pathological gambling: betting on GPs' beliefs and attitudes. Biomed Res Int. (2014) 2014:360585. 10.1155/2014/360585 25243136 PMC4160611

[27] Suomi A Lucas N Dowling N Delfabbro P. Parental problem gambling and child wellbeing: systematic review and synthesis of evidence. Addict Behav. (2022) 126:107205. 10.1016/j.addbeh.2021.107205 34890890

[28] Stefanovics EA Potenza MN Pietrzak RH. Gambling in a national U.S. veteran population: prevalence, socio-demographics, and psychiatric comorbidities. J Gambl Stud. (2017) 33:1099–120. 10.1007/s10899-017-9678-2 28293767

[29] Grall-Bronnec M Sauvaget A Perrouin F Leboucher J Etcheverrigaray F Challet-Bouju G . Pathological gambling associated with aripiprazole or dopamine replacement therapy: do patients share the same features? A review. J Clin Psychopharmacol. (2016) 36:63–70. 10.1097/JCP.0000000000000444 26658263 PMC4700874

