# Supplementary material for: GamReg Sweden—Protocol for a systematic cohort data collection for improved clinical knowledge in specialized gambling disorder treatment
Source: Front Psychiatry. 2022 Sep 12;13:894532. doi: 10.3389/fpsyt.2022.894532 (PMC9510646; doi:10.3389/fpsyt.2022.894532)
Supplement: Supplementary file 1 [file Data_Sheet_1.PDF]

Supplementary material:

Quality register GamReg Sweden – variable list

Name of unit ____________________________________

Type of unit

- ☐ municipality
- ☐ health care (public region or private)

Personal identity number _______________________________________

Gender

- ☐ male
- ☐ female
- ☐ other (transgender, non-binary)

Date of start of present treatment contact _____________________

How was the patient referred to the present unit?

- ☐ self-referral
- ☐ referred from social services
- ☐ referred from primary care unit, occupational primary care unit, or similar
- ☐ referred from psychiatric health care
- ☐ referred from addiction health care
- ☐ referred from residential treatment facility
- ☐ referred from NGO
- ☐ referred from national helpline
- ☐ referred from criminal justice system

Does the present treatment contact partly of fully involve problematic gaming behavior (not primarily involving money)?

☐ yes ☐ no

If the treatment contact involves gambling for money: type of problematic gambling (several can be reported):

- ☐ casino online
- ☐ land-based casino or 'restaurant casino'
- ☐ horse race betting online

☐ horse race betting land-based
☐ sports betting – live betting
☐ sports betting – non-live betting
☐ online poker
☐ poker – land-based
☐ electronic gambling machines – land-based
☐ bingo – online
☐ bingo – on tv, in physical venue, ’car bingo’
☐ lotteries – scratch tickets, internet tickets
☐ number games – ’Lotto’, ’Keno’, ’Joker’
☐ stocks, options, other stock market values
☐ gambling for money as part of a video game
☐ other gambling

If the treatment contact involves problem gaming: type of problematic gaming (several can be reported):

☐ single player
☐ multi player
☐ massively multiplayer online game
☐ unknown / other

How old was the patient when the problem started?

____

Main source of income

☐ work
☐ studying
☐ unemployment benefit
☐ retired
☐ sick leave
☐ social welfare support
☐ other (report in free text: ______________________)

Has the patient ever been subject to the national enforcement agency?

☐ yes ☐ no ☐ unknown

Has the patient, at any time during the past 12 months, had

any contact with health care services (psychiatry, primary care, etc) due to poor mental health?

☐ yes ☐ no ☐ unknown

any contact with social services (other than if the registering unit belongs to social services, and other than social welfare support)?

☐ yes ☐ no ☐ unknown

alcohol problems, for which the patient has sought treatment or felt a need to seek treatment for?

☐ yes ☐ no ☐ unknown

Problems related to prescription drugs or illicit drugs, for which the patient has sought treatment or felt a need to seek treatment for?

☐ yes ☐ no ☐ unknown

Has the patient ever been hospitalized due to mental health problem or due to addictive behaviors?

☐ yes ☐ no ☐ unknown

Known suicidal act (suicide attempt) ever?

☐ yes ☐ no ☐ unknown

Has the patient ever reported being a victim of severe physical, psychological, or sexual violence, at any time?

☐ yes ☐ no ☐ unknown

Does the patient have prescribed drug treatment for ADD/ADHD?

☐ yes ☐ no ☐ unknown

Does the patient live with a partner/spouse?

☐ yes ☐ no ☐ unknown

Does the patient have children under the age of 18 years?

☐ yes ☐ no ☐ unknown

Does the patient live with children under the age of 18 years?

☐ yes ☐ no ☐ unknown

Has the child, during the treatment contact, received any counselling or support?

☐ yes ☐ no ☐ unknown

Treatment which the patient has received at this unit (several can be reported):

□ CBT individually
□ CBT in group
□ CBT online
□ motivational interviewing
□ relapse prevention
□ 12-step treatment
□ prescribed drug treatment for gambling disorder with naltrexone
□ prescribed drug treatment for gambling disorder with nalmefene/Selincro
□ prescribed drug treatment for gambling disorder with both naltrexone and nalmefene/Selincro
□ residential treatment
□ psycho-dynamic therapy
